# A novel photic entrainment mechanism for the circadian clock in an insect: involvement of *c-fos* and *cryptochrome*s

**DOI:** 10.1186/s40851-018-0109-8

**Published:** 2018-09-18

**Authors:** Yuki Kutaragi, Atsushi Tokuoka, Yasuaki Tomiyama, Motoki Nose, Takayuki Watanabe, Tetsuya Bando, Yoshiyuki Moriyama, Kenji Tomioka

**Affiliations:** 1 Graduate School of Natural Science and Technology, Okayama University, Okayama, 700-8530 Japan; 2 Research Institute for Electronic Science, Hokkaido University, Sapporo, 060-0811 Japan; 3 Graduate School of Medicine, Dentistry and Pharmaceutical Sciences, Okayama University , Okayama, 700-8558 Japan; 4 Department of Natural Sciences, Kawasaki Medical School, Matsushima 577, Kurashiki, 701-0192 Japan

**Keywords:** *C-fos*, Circadian clock, Clock gene, *Cryptochrome*, Insect, Photic entrainment

## Abstract

**Background:**

Entrainment to the environmental light cycle is an essential property of the circadian clock. Although the compound eye is known to be the major photoreceptor necessary for entrainment in many insects, the molecular mechanisms of photic entrainment remain to be explored.

**Results:**

We found  that *cryptochrome*s (*cry*s) and *c-fos* mediate photic entrainment of the circadian clock in a hemimetabolous insect, the cricket *Gryllus bimaculatus*. We examined the effects of RNA interference (RNAi)-mediated knockdown of the *cry* genes, *Gb’cry1* and *Gb’cry2*, on photic entrainment, and light-induced resetting of the circadian locomotor rhythm. *Gb’cry2* RNAi accelerated entrainment for delay shifts, while *Gb’cry1/ Gb’cry2* double RNAi resulted in significant lengthening of transient cycles in both advance and delay shifts, and even in entrainment failure in some crickets. Double RNAi also strongly suppressed light induced resetting. The *Gb’cry-*mediated phase shift or resetting of the rhythm was preceded by light-induced *Gb’c-fosB* expression. We also found that *Gb’c-fosB*, *Gb’cry2* and *Gb’period* (*Gb’per*) were likely co-expressed in some optic lobe neurons.

**Conclusion:**

Based on these results, we propose a novel model for photic entrainment of the insect circadian clock, which relies on the light information perceived by the compound eye.

**Electronic supplementary material:**

The online version of this article (10.1186/s40851-018-0109-8) contains supplementary material, which is available to authorized users.

## Background

The circadian clock is an endogenous, highly conserved timing mechanism in animals that is used to anticipate and adapt to daily environmental changes [1]. The oscillatory mechanism of insect clocks consists of interlinked transcriptional and translational feedback loops [2–4]. The major players in the loops are so called ‘clock genes’, including *period* (*per*), *timeless* (*tim*), *Clock* (*Clk*), and *cycle* (*cyc*). It is generally thought that the products of *Clk* and *cyc* genes heterodimerize to form a CLOCK (CLK)/CYCLE (CYC) complex, which activates transcription of *per* and *tim* in the late day to early night, and PERIOD (PER) and TIMELESS (TIM) proteins form a heterodimer that then inhibits CLK/CYC transcriptional activity later at night [2, 3]. This negative feedback is thought to produce an approximately 24 h rhythm. There is an additional loop producing rhythmic expression of either *Clk* or *cyc* [4]. This oscillatory mechanism includes *vrille* (*vri*) and *Par domain protein 1* (*Pdp1*) for *Clk* [5, 6], and *ecdysone induced protein 75* (*E75*) and * hormone receptor 3* (*HR3*) for *cyc* [7].

An essential property of the clock is the ability to synchronize with daily environmental cycles, with sunlight as the most important time cue. The mechanism for this synchronization, or entrainment, is best understood in *Drosophila*, which uses CRYPTOCHROME (dCRY or CRY1) as a photoreceptor molecule. CRY1 is a flavin-based blue light receptor, and is known to be expressed in a limited number of clock neurons [8], where it leads to TIM degradation in a light dependent manner [9, 10], and resets the clock [11, 12]. However, many insects possess another type of CRY, CRY2 [13, 14], which is more similar to mammalian CRYs. The role of mammalian CRYs is not completely understood. Certain lines of evidence suggest they are involved in the core oscillatory mechanism, working together with PER to repress transcriptional activity of CLK and BRAIN AND MUSCLE ARNT LIKE 1 (BMAL1, the mammalian homologue of CYC) complex [15, 16]. However, some studies have shown that mammalian CRY also plays a role as a photoreceptor, and is involved in photic entrainment of the suprachiasmatic nucleus (SCN) or peripheral clocks [17, 18]. In insects, CRY2 is believed to be involved in the core clock oscillatory mechanism [14], mainly based on assays using cultured cell systems. The role of CRY2 is yet to be explored in vivo.

In the present study, we investigated the role of *cry1* and *cry2* genes in photic entrainment of the circadian clock in the cricket, *Gryllus bimaculatus*. In this cricket, photic entrainment solely depends on the compound eye [19, 20], and the major circadian photoreceptor molecule in the compound eye is opsin-Long Wavelength (*Gb’*OpLW) [21, 22]. We have previously shown that resetting of the clock by the extension of the light phase during the early subjective night includes transcriptional regulation of clock genes, with *Pdp1* as the first responder to light [22]. However, the reset mechanism in other situations, e.g. in free-running conditions or during the night, remains unknown. We have recently shown that the cricket genome includes two *cry* genes, which are involved in the oscillatory machinery of its internal clock [23]. Unlike other insects, *Gb’cry2* has several transcriptional variants that form a feedback loop in a specific combination with the isoforms and *Gb’cry1* [23]. Our RNAi experiments reveal for the first time that re-entrainment to shifted light cycles was rather accelerated by reduced expression of *Gb’cry2,* but severely disrupted by *Gb’cry1* and *Gb’cry2* double knock-down. We also show that *Gb’c-fosB* is involved in the photic entrainment pathway. Based on these results, we propose a novel model of photic entrainment of the insect circadian clock, which furthers our understanding of the insect circadian system.

## Materials and methods

### Experimental animals

Eighth instar nymphs and adult males of the cricket, *Gryllus bimaculatus*, were used. They were purchased or obtained from a laboratory colony maintained under standard environmental conditions, with a lighting regimen of alternating 12 h light and 12 h darkness (LD 12:12; light: 0600–1800; Japan standard time, JST) and at a constant temperature of 25 ± 0.5 °C. They were fed laboratory chow and water.

### Measurement of mRNA levels

The mRNA levels of *Gb’cry1* (GenBank/EMBL/DDBJ Accession No. LC202047), *Gb’cry2* (LC202053), *Gb’c-fosA* (*fra-A*, LC215243), *Gb’c-fosB* (*fra-B*, LC215244), and *Gb’Pdp1* were measured by quantitative real-time polymerase chain reaction (qPCR) [22]. Total RNA was extracted and purified from six adult male optic lobes with TRIzol Reagent (Invitrogen, Carlsbad, CA, USA), and treated with DNase I to remove contaminating genomic DNA. About 500 ng total RNA from each sample was reverse transcribed with random 6mers using PrimeScript RT reagent kit (TaKaRa, Shiga, Japan). qPCR was performed in the Mx3000P real-time PCR system (Stratagene, La Jolla, CA, USA) using FastStart Universal SYBR Green Master (Roche, Tokyo, Japan) including SYBR Green, with primers listed in Additional file 1: Table S1. We used *Gb’rpl18a* (GenBank/EMBL/DDBJ Accession No. DC448653) as an internal reference gene. Quantification was based on a standard curve obtained with known amounts of template DNA. The results were analyzed using the instrument vendor-associated software. The values were normalized with those of *Gb’rpl18a* at each time point. Results of 3–8 independent experiments were used to calculate the mean ± SEM.

### RNAi

Double stranded RNA (dsRNA) for *Gb’cry1*, *Gb’cry2*, *Gb’c-fosA*, *Gb’c-fos* (for targeting both *Gb’c-fosA* and *Gb’c-fosB*), *Gb’opsin-long wavelength* (*Gb’opLW*) (GenBank/EMBL/DDBJ accession No. LC004297), *Gb’opsin-blue* (*Gb’opBlue*) (LC004296), and *DsRed2* derived from a coral species (*Discosoma* sp.), were synthesized using MEGAscript High Yield Transcription Kit (Ambion, Austin, TX, USA). For *Gb’cry1*, *Gb’cry2*, *Gb’c-fos*, *Gb’c-fosA*, *Gb’opLW*, and *Gb’opBlue,* template cDNA fragments for in vitro transcription were amplified by PCR from the cricket brain cDNA library using ExTaq DNA polymerase (TaKaRa). Primers tagged with T7 or T3 promoter sequences were used for PCR amplification (primer sequences are listed in Additional file 1: Table S1). For *DsRed2* dsRNA, a *DsRed2* cDNA fragment was amplified from pDsRed2-N1 (Clontech, Mountain View, CA, USA) with primers listed in Additional file 1: Table S1. Amplified fragments were purified with phenol/chloroform and precipitated with ethanol. RNA was synthesized from each of these cDNA fragments using T7 or T3 RNA polymerase. Synthesized RNA was extracted with phenol/chloroform, and suspended in 50 μl TE buffer after isopropanol precipitation. The yield and quality of RNA was assessed by spectrophotometer (Genequant Pro, Amersham Bioscience, Piscataway, NJ, USA), and equal amounts of sense and antisense RNAs were then mixed. The RNA mixture was denatured for 5 min at 100 °C and annealed by a gradual cooling to room temperature (25 °C). After ethanol precipitation, the obtained dsRNA was suspended in UltraPure DNase/RNase-Free Distilled Water (Invitrogen) and adjusted to a final concentration of 20 μM. The dsRNA solution was stored at − 80 °C until use. 760 nl of dsRNA solution was injected with Nanoliter Injector (WPI, Sarasota, FL, USA) into the abdomen of 8th instar nymphs or adult crickets anesthetized with CO_2_.

### In situ hybridization

The adult male heads were collected at Zeitgeber time 18 (ZT18: ZT 0 and ZT 12 correspond to light-on and light-off, respectively), fixed for 24 h at 4 °C with PFA solution (4% paraformaldehyde in phosphate buffered saline), dehydrated by a series of butyl alcohol and ethanol, and embedded in paraffin. Tissues were sectioned at 6 μm and mounted on MAS-GP type A coated slides (Matsunami Glass, Osaka, Japan). In situ hybridization (ISH) was performed using ViewRNA ISH Tissue Assay (Affymetrix, Santa Clara, CA) following the manufacturer’s protocol. In brief, tissue sections were subjected to xylene deparaffinization followed by ethanol dehydration. To unmask the RNA targets, deparaffinized sections were incubated in pretreatment buffer at 90–95 °C for 10 min and digested with protease (1:100 dilution) at 40 °C for 10 min, followed by fixation with 10% neutral buffered formalin at room temperature for 5 min. Unmasked tissue sections were subsequently hybridized with the ViewRNA probe set (1:50 dilution) for 2 h at 40 °C, followed by series of post-hybridization washes. The ViewRNA probes used for detecting *Gb’per* (Accession No. AB375516) and *Gb’cry2* were designed and synthesized by Affymetrix, covering 1027–2016 and 1548–2669 base region, respectively. A non-probe sample was utilized as a negative control. Signal amplification was achieved via a series of sequential hybridizations and washes according to the manufacture’s protocol. Signals for *Gb’per* and *Gb’cry2* were detected with Fast Red or Fast Blue substrate, respectively. Slides were post-fixed in 10% neutral buffered formalin, mounted in Dako Ultramount mounting medium (Dako, Carpinteria, CA), observed and photographed using light microscopy (BZ-X700, KEYENCE, Osaka, Japan).

### In situ RT-PCR

Heads were collected at ZT21 from adult males that were exposed to light for 1 h from ZT20. They were fixed in 4% PFA solution for 24 h at 4 °C, dehydrated by a series of butyl alcohol and ethanol, and embedded in paraffin. Tissues were sectioned at 6 μm and mounted on MAS-GP type A coated slides. The sections were pretreated with 1 U/μl DNase I (TaKaRa) in DNase buffer containing 2 U/μl RNase inhibitor (TaKaRa) at 37 °C overnight. Following the DNase I treatment, the sections were washed with RNase-free PBS and RNase-free water. One-step in situ RT-PCR was performed using RT-PCR Quick Master Mix (TOYOBO, Osaka, Japan) in a thermal cycler (Mastercycler, Eppendorf) with in situ Adapter. Final concentration of the reaction mixture was as follows: 1× RT-PCR Quick Master Mix, 2.5 mM Mn(OAc)_2_, 0.2 μM of forward and reverse primers for *Gb’c-fosB* (described in Additional file 1: Table S1), 40 U/μl RNase inhibitor (TaKaRa), 1.2 μl/100 μl of 1 mM digoxigenin (DIG)-11-dUTP (Roche) and relevant amount of RNase free water. The cDNA was synthesized at 60 °C for 30 min. PCR amplification consisted of an initial denaturation step of 94 °C for 1 min, followed by 20 reaction cycles of denaturation (94 °C, 30 s), annealing (60 °C, 30 s), and extension (72 °C, 1 min), then by termination with a final extension reaction at 72 °C for 7 min. The sections were fixed with 4% PFA solution for 10 min at 4 °C and washed in 0.1× standard saline citrate and washing buffer. After a blocking step, the sections were incubated with alkaline-phosphatase-conjugated sheep anti-digoxigenin Fab antibody (Roche). DIG-labeled PCR products were detected with 4-nitro blue tetrazolium chloride (Roche) and 5-bromo-4-chloro-3-indolyl phosphate (Roche) by incubating the sections for the relevant length of time. The sections were mounted, observed and photographed using light microscopy (BZ-X700, KEYENCE).

### Behavioral analysis

Locomotor activities were recorded according to Moriyama et al. [24]. Briefly, the final instar nymphs or adult crickets were individually housed in a transparent plastic box (18 × 9 × 4.5 cm) with a rocking substratum. The number of substratum rocks was recorded every 6 min by a computerized system. Food and water were provided ad libitum. The actographs were placed in an incubator (MIR-153, Sanyo Biomedica, Osaka, Japan) with constant temperature at 25 ± 0.5 °C, and light was introduced by a cool white fluorescent lamp connected to an electric timer. The light intensity was 600–1000 lx at the animal’s level, varying with proximity to the lamp. The raw data were displayed as conventional double-plotted actograms to judge activity patterns, and statistically analyzed by the chi-square periodogram [25] with Actogram J (http://actogramj.neurofly.de/) [26]. If a peak of the periodogram appeared above the 0.05 confidence level, the power value (height of the peak above the confidence level) was greater than or equal to 10, and the width of the peak was greater than or equal to 2, the period for the peak was designated as statistically significant [27].

The magnitude of phase shifts caused by a light pulse or a light phase extension was estimated by fitting a regression line to daily activity onsets at steady-state free-running in constant darkness (DD) after light treatments. The phase of the free-running rhythm was determined on the day of light treatment by extrapolating the regression line. The same value was obtained for the control crickets, which received the same treatments but were transferred to DD without light treatment. The magnitude of phase shift of an animal caused by light treatments was estimated by subtracting the average value of the control group from the value of each light-treated animal.

### Statistical analysis

One-way analysis of variance (ANOVA) followed by a post-hoc Tukey-Kramer test was used to compare differences in the mean mRNA levels between the different time points, or in the means of magnitudes of phase shifts between groups with various treatments. To compare the means of two groups, t-test was used. In all statistical tests, the significance level was set at α = 0.05.

## Results

### *Gb’cry1* and *Gb’cry2* play an important role in photic entrainment

**Fig. 1:**
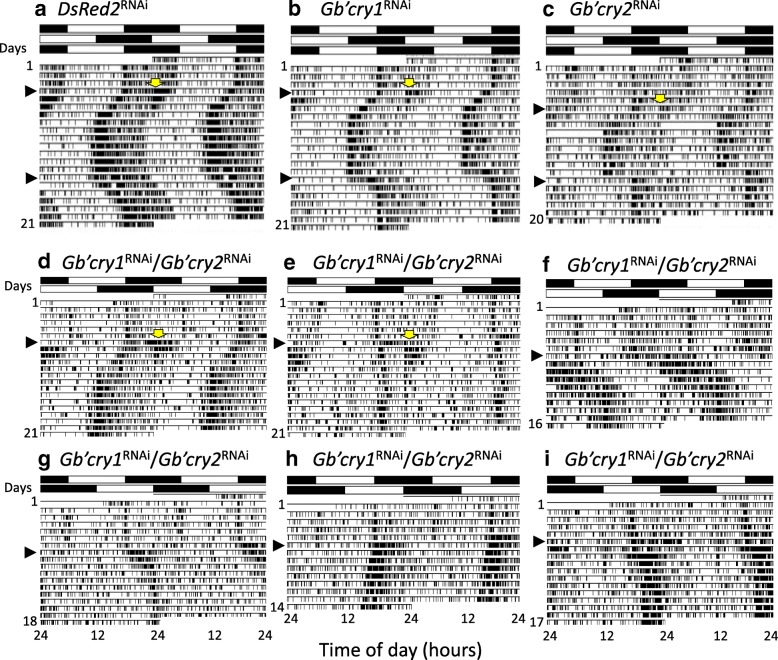
Entrainment of the locomotor rhythm to LD12:12 in *DsRed2*^RNAi^ (**a**), *Gb’cry1*^RNAi^ (**b**), *Gb’cry2*^RNAi^ (**c**), and *Gb’cry1*^RNAi^*/Gb’cry2*^RNAi^ crickets (*Gryllus bimaculatus*) (**d**-**i**). Light cycles were advanced or delayed by 6 h on the day indicated by an arrowhead on the left side of the actograms. White and black bars above the actograms indicate light (white) and dark (black) cycles. Yellow arrows indicate the light induced activity at light-on after a 6 h phase advance of the light cycle. Transient cycles were shorter in *Gb’cry1*^RNAi^ and *Gb’cry2*^RNAi^ crickets than in the control (*DsRed2*^RNAi^) crickets, but were longer in *Gb’cry1*^RNAi^*/Gb’cry2*^RNAi^ crickets. Some of the *Gb’cry1*^RNAi^*/Gb’cry2*^RNAi^ crickets (**e**, **h, i**) apparently lost entrainability

We examined the effects of *Gb’cry1*^RNAi^ and *Gb’cry2*^RNAi^ on light entrainment of the locomotor rhythm (Fig. 1). We first confirmed that the crickets were entrained to LD12:12. The onset of their nocturnal activity occurred slightly before light-off for both *Gb’cry1*^RNAi^ and *Gb’cry2*^RNAi^, and their Ψ values were similar to that of *DsRed2*^RNAi^ control crickets (Table 1). When the light cycle was advanced or delayed by 6 h, control crickets treated with ds*DsRed2* re-synchronized to the shifted LD, with transient cycles of approximately four days for both shift directions (Table 2). Although *Gb’cry1*^RNAi^ and *Gb’cry2*^RNAi^ crickets also re-synchronized to the shifted LD cycles, the transients tended to be a little shorter than those of the control crickets; however, only the delay shifts of *Gb’cry2*^RNAi^ crickets were statistically significant (Table 2). Although some of the *Gb’cry2*^RNAi^ crickets showed instantaneous entrainment to a delayed LD (Fig. 1c), we have to examine whether this is true entrainment or a result of strong negative masking of light. An intense bout of activity occurred at light-on during the transient cycles in advance shifts (Fig. 1a-c). Treatment with dsRNA of *Gb’cry2* significantly reduced the light-induced responses (Fig. 1c, Additional file 2: Figure S1). The Ψ after the shifts was again close to that of the *DsRed2*^RNAi^ controls (Table 1).

**Table 1:** Effects of *Gb’cry1* and *Gb’cry2* RNAi on the phase relationship between light-off and activity onset. Different lower case letters indicate that values are significantly different (ANOVA followed by Tukey-test, *P* < 0.05). Ψ_advance_ and Ψ_delay_ indicate reestablished phase relationship after 6 h phase advance or delay of LD cycles, respectively. Numbers in parenthesis indicate number of animals used

Treatment	Ψ_original_	Ψ_advance_	Ψ_delay_
ds*DsRed2*	0.51 ± 0.46 h (28)	0.68 ± 0.56^a^ h (18)	0.92 ± 0.82^ab^ h (13)
ds*Gb’cry1*	0.60 ± 0.51 h (35)	0.69 ± 0.60^a^ h (28)	0.73 ± 0.66^ab^ h (21)
ds*Gb’cry2*	0.52 ± 0.78 h (15)	0.56 ± 0.74^a^ h (16)	0.30 ± 0.28^a^ h (14)
ds*Gb’cry1/*ds*Gb’cry2*	0.87 ± 0.84 h (45)	1.69 ± 1.60^b^ h (14)	1.02 ± 0.76^b^ h (18)

**Table 2:** Effects of *Gb*’*cry1* and *Gb’cry2* RNAi on re-entrainment of the circadian locomotor rhythms in *Gryllus bimaculatus*. Different lower case letters indicate that values are significantly different (ANOVA followed by Tukey-test, *P* < 0.05)

Treatment	N	Re-entrained animals (%)	Transient cycles (Mean ± SD; days)
6 h advance			
ds*DsRed2*	22	100	4.0 ± 0.84^a^
ds*Gb’cry1*	30	100	3.57 ± 0.72^a^
ds*Gb’cry2*	16	100	3.27 ± 1.22^a^
ds*Gb’cry1/*ds*Gb’cry2*	16	94	6.8 ± 1.52^b^
6 h delay			
ds*DsRed2*	14	100	4.0 ± 0.39^a^
ds*Gb’cry1*	16	100	3.25 ± 1.0^a^
ds*Gb’cry2*	13	100	1.15 ± 0.38^b^
ds*Gb’cry1/*ds*Gb’cry2*	18	89	5.56 ± 1.59^c^

We then tested the effect of *Gb’cry1* and *Gb’cry2* double knock-down on photic entrainment (Fig. 1d-i). For both advance and delay shifts, some fraction of the treated crickets could not synchronize within 14 days, as exemplified in Fig. 1e, h, and i. The loss of entrainment was observed in 6% (1/16) and 11% (2/18) of crickets, for advance and delay shifts, respectively. Even in the re-entrained crickets, the transient cycles were significantly greater than in control crickets, with cycles of 6.8 ± 1.52 days and 5.56 ± 1.59 days for advance and delay shifts, respectively (Table 2), and the time course and direction of re-entrainment were variable, with some crickets responding to a 6 h advance shift by synchronizing with delay shifts (Fig. 1f). This variability may be related to the wide variation of the free-running period in the double RNAi crickets [23]. Interestingly, some crickets showed a rhythm splitting, where a component re-synchronized with a gradual advance while the other component did not respond and stayed almost at the same phase (Fig. 1e). The established final phase-relationship with LD was often abnormal in the double RNAi crickets, leading to greater Ψ values (Table 1), although the difference was significant only after advance shifts. Similar to *Gb’cry2*^RNAi^ crickets, the light-induced activity during advance shifts was significantly reduced than that of the control crickets (Additional file 2: Figure S1).

### Effects of *Gb’cry1*^RNAi^ and *Gb’cry2*^RNAi^ on phase responses to a light pulse

**Fig. 2:**
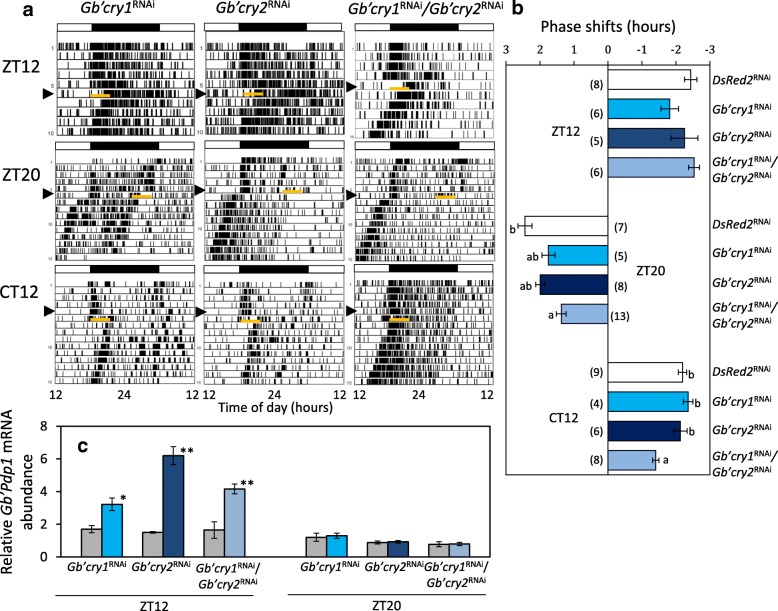
Effects of RNAi of *Gb’cry* genes on light-induced phase shifts of circadian locomotor rhythm (**a**, **b**) and on light-induced *Gb’Pdp1* expression (**c**) in the cricket *Gryllus bimaculatus*. **a** Phase shifts of locomotor rhythm caused by a 3 h light phase extension at ZT12 and a 3 h light exposure at ZT20 or CT12. Orange bars indicate a 3 h light phase extension or a light pulse. Black and white bars indicate dark and light phases, respectively. Arrow heads indicate the day of transfer to constant darkness. **b** Average phase shifts caused by a light phase extension at ZT12 or by a 3 h light pulse given at ZT20 or CT12. Error bars indicate SEM. Number in parenthesis indicates the number of crickets used. Different lower case letters indicate that values are significantly different (ANOVA followed by Tukey-Krammer test, *P* < 0.05). **c** Effects of light exposure on *Gb’Pdp1* mRNA levels at ZT12 or ZT20. Colored and grey bars indicate the results with and without 60 min light exposure, respectively. Light phase extension at ZT12 induced significant upregulation of *Gb’Pdp1* expression (t-test, *P* < 0.05) in all *Gb’cry*^RNAi^ crickets, while light exposure at ZT20 had no effect

We examined the effects of *Gb’cry1*^RNAi^ and *Gb’cry2*^RNAi^ treatment on phase-shifts of the locomotor rhythm caused by a 3 h light pulse given at late night (ZT20) or early subjective night (Circadian time (CT) 12: CT 0 and CT 12 correspond to subjective dawn and subjective sunset, respectively) after a transfer to DD. A 3 h light exposure given at ZT20 caused a phase advance by 2.44 ± 0.54 h (*n* = 7) in *DsRed2*^RNAi^ crickets. The *Gb’cry1*^RNAi^ and *Gb’cry2*^RNAi^ crickets showed advance shifts (1.75 ± 0.47 h, *n* = 5; 1.99 ± 0.37 h, *n* = 8, respectively, Fig. 2) with the magnitude slightly less than that of the control, but the difference was not significant. In *Gb’cry1/Gb’cry2* double RNAi crickets, the shift was 1.37 ± 0.53 h (*n* = 13), and was significantly smaller than that of the control (Fig. 2). A 3 h light pulse given at CT12 induced delay shifts in *Gb’cry1*^RNAi^ and *Gb’cry2*^RNAi^ crickets, with the magnitude similar to that of *DsRed2*^RNAi^ control crickets, while in *Gb’cry1*^RNAi^*/Gb’cry*2^RNAi^ crickets*,* the magnitude was − 1.41 ± 0.26 h (n = 8), which was significantly smaller than that of the control (− 2.20 ± 0.37 h, *n* = 9) (Fig. 2a, b).

We also examined the effects of a 3 h extension of light phase (ZT12–15) on the phase of free-running locomotor rhythms in the ensuing DD. There were no significant effects of single or double dsRNA treatment of *Gb’cry* genes on the magnitude of the phase shifts (Fig. 2a, b).

### Effects of *Gb’cry1* and *Gb’cry2* knock-down on photic responses of *Gb’Pdp1*

We next examined the effects of *Gb’cry1*^RNAi^ and *Gb’cry2*^RNAi^ treatment on light-induced *Gb’Pdp1* upregulation, which is the first responder to light phase extension at early subjective night [22]. We measured the mRNA levels of *Gb’Pdp1* 1 h after light phase extension starting at ZT12, or 1 h after light exposure starting at ZT20*.*

In all RNAi crickets, *Gb’Pdp1* was upregulated 1 h after light-on at ZT12 in comparison to the control without light exposure, while no significant changes were observed in *Gb’Pdp1* levels when a light pulse was given at ZT20 (Fig. 2c). The results were similar to those observed in untreated crickets [22], suggesting that *Gb’cry*s are not upstream components of the transcription-dependent entrainment pathway.

### *Gb’c-fosB* is involved upstream of *Gb’cry*s

**Fig. 3:**
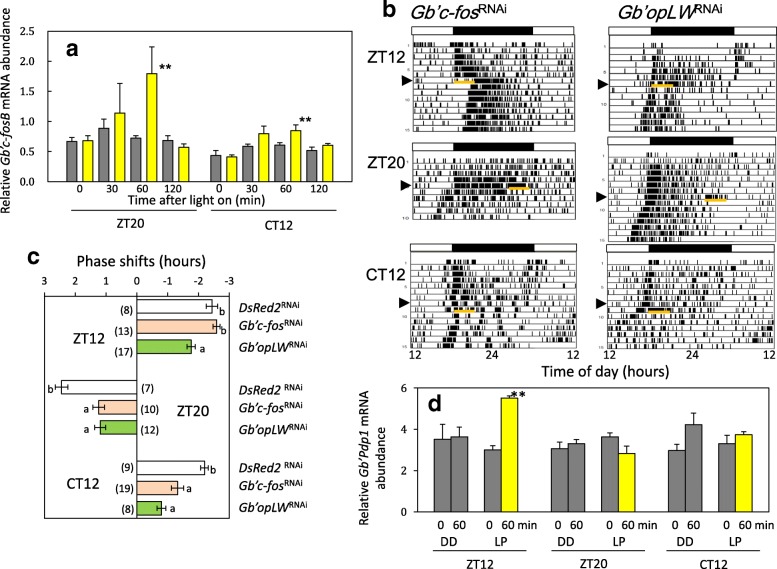
Changes of *Gb’c-fosB* mRNA levels caused by light exposure (**a**), effects of RNAi of *Gb’c-fos* and *Gb’opLW* genes on the light-induced phase shifts of locomotor rhythms (**b**, **c**), and effects of *Gb’c-fos*^RNAi^ on the light induced *Gb’Pdp1* expression (**d**) in the cricket *Gryllus bimaculatus*. **a** *Gb’c-fosB* was upregulated by light exposure at ZT20 and CT12, and a statistically significant difference was evident after 60 min of exposure (**P < 0.01, t-test). The effect was greater in the late night (ZT20). Yellow and grey bars indicate the results with and without light exposure, respectively. **b** Phase shifts of locomotor rhythm caused by a 3 h light phase extension at ZT12 or 3 h light exposure at ZT20 or CT12 (orange bars). Black and white bars indicate dark and light phases, respectively. Arrow heads indicate the day of transfer to constant darkness. **c** Average phase shifts caused by a light phase extension at ZT12 or by a 3 h light pulse given at ZT20 or CT12. Error bars indicate SEM. Different lower-case letters indicate that values are significantly different (ANOVA followed by Tukey-Kramer test, *P* < 0.05). *Gb’c-fos*^RNAi^ suppressed the light-induced phase shifts at ZT20 and CT12 but not at ZT12, and *Gb’opLW*^RNAi^ strongly suppressed them in all cases (ANOVA followed by Tukey-Kramer test, P < 0.01). **d** Effects of light exposure on *Gb’Pdp1* mRNA levels at ZT12, ZT20, and CT12 in *Gb’c-fos*^RNAi^ crickets. Yellow and grey bars indicate the results with and without 60 min light exposure, respectively. Light phase extension at ZT12 induced significant upregulation of *Gb’Pdp1* expression (**P < 0.01, t-test) in *Gb’c-fos*^RNAi^ crickets, while no apparent effect was observed following light exposure at ZT20 and CT12

Since a bZip transcription factor gene, *c-fos,* is known to be up-regulated by light exposure in mammalian circadian clocks [28], we examined whether it also responded to light in crickets. *G. bimaculatus* has two isoforms of *c-fos*: *Gb’c-fosA* (*fra-A*, LC215243) and *Gb’c-fosB* (*fra-B*, LC215244), which arise from alternative promoter usage or alternative splicing from a single locus. We thus tested the effects of a 3 h light exposure at ZT20 and CT12 on their expression levels. *Gb’c-fosB* was significantly upregulated following light exposure, increasing from 30 min after light-exposure, but increasing significantly after 60 min (Fig. 3a), and then decreasing to basal levels after 120 min.

We then examined the effect of *Gb’c-fos*^RNAi^ on the light-induced resetting of the circadian locomotor rhythm. *Gb’c-fosA* and *Gb’c-fosB* were both targeted because their nucleotide sequences are mostly identical, and we could not make a specific knock-down of *Gb’c-fosB*. The dsRNA treatment effectively knocked down the *Gb’c-fosB* mRNA levels to approximately 25% of that of the *DsRed2*^RNAi^ controls (Additional file 3: Figure S2). The *Gb’c-fos*^RNAi^ had no significant effect on the locomotor rhythm; the treated crickets showed a rhythm synchronized to LD cycles and free-running in DD (Fig. 3b). There was no significant difference in free-running period in DD between *DsRed2*^RNAi^ crickets (23.70 ± 0.38 [mean ± SD] h, *n* = 9) and *Gb’c-fos*^RNAi^ crickets (23.81 ± 0.30 h, *n* = 8) which were transferred directly to DD. A 3 h light pulse given at CT12 or ZT20 delayed or advanced the rhythm in *Gb’c-fos*^RNAi^ by − 1.32 ± 0.86 h (*n* = 19) and 1.24 ± 0.59 h (*n* = 10), respectively, but the magnitude was significantly smaller than that of *DsRed2*^RNAi^ control crickets (Fig. 3b, c). We also used dsRNA specific for *Gb’c-fosA,* and found no significant effects on advance shifts caused by a 3 h light pulse at ZT20 (Additional file 4: Figure S3). The suppression of phase-shifts by *Gb’c-fos*^RNAi^ was therefore apparently caused by knockdown of *Gb’c-fosB*. However, no suppression was observed when light phase was extended by 3 h at ZT12 (Fig. 3b, c).

Following RNAi of *Gb’opLW*, light induced phase shifts were significantly reduced in all light treatments (Fig. 3b, c), being consistent with previous observations [21, 22]. We further examined the effects of *Gb’c-fos* knock-down on *Gb’Pdp1* levels in response to light phase extension. In *Gb’c-fos*^RNAi^ crickets, *Gb’Pdp1* mRNA levels were up-regulated when the light phase was extended at ZT12, while no significant changes were observed when light was given at ZT20 or CT12 (Fig. 3d), similar to control crickets [22], suggesting that the *Pdp1*-dependent entrainment pathway is independent of the *c-fos* pathway.

**Fig. 4:**
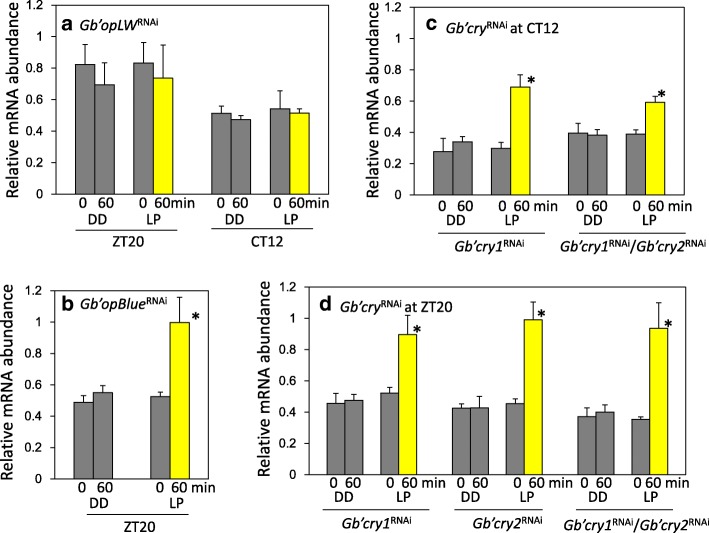
Effects of RNAi of *Gb’opLW* (**a**), *Gb’opBlue* (**b**), and *Gb’cry* genes (**c**, **d**) on light induced *Gb’c-fosB* mRNA expression in the cricket *Gryllus bimaculatus*. Gray and yellow columns indicate samples kept in darkness or exposed to light for 60 min, respectively. DD, kept in darkness; LP, exposed to light. Bars indicate SEM. *N* = 3 or 4. **a** *Gb’opLW*^RNAi^ suppressed light-induced upregulation of *Gb’c-fosB*. **b-d** RNAi of *Gb’opBlue* or *Gb’cry* genes had no effects on the light-induced upregulation of *Gb’c-fosB* at ZT20 and CT12. *P < 0.05, t-test

We then examined the effects of *Gb’opLW*^RNAi^ on *Gb’c-fosB* levels, since *Gb’*OpLW is the major photoreceptor for photic entrainment in this cricket [21, 22]. We observed no significant changes in *Gb’c-fosB* levels following light exposure at CT12 and ZT20 in *Gb’opLW*^RNAi^ crickets (Fig. 4a), while *Gb’c-fosB* was significantly upregulated in *Gb’opBlue*^RNAi^ crickets 1 h after light exposure at ZT20 (Fig.4b). This suggests that the signal from *Gb’*OpLW is required for the *Gb’c-fosB* dependent entrainment pathway.

We then examined the effects of *Gb’cry* knock-down on *Gb’c-fosB* mRNA levels. We found that *Gb’cry1* or *Gb’cry2* single knock-down or double knock-down did not affect light-induced upregulation of *Gb’c-fosB* at CT12 or ZT20 (Fig. 4c, d), suggesting that *Gb’cry*s are downstream of *Gb’c-fosB*.

### *Gb’cry2*, *Gb’per*, and *Gb’c-fosB* are expressed in the optic lobe

**Fig. 5:**
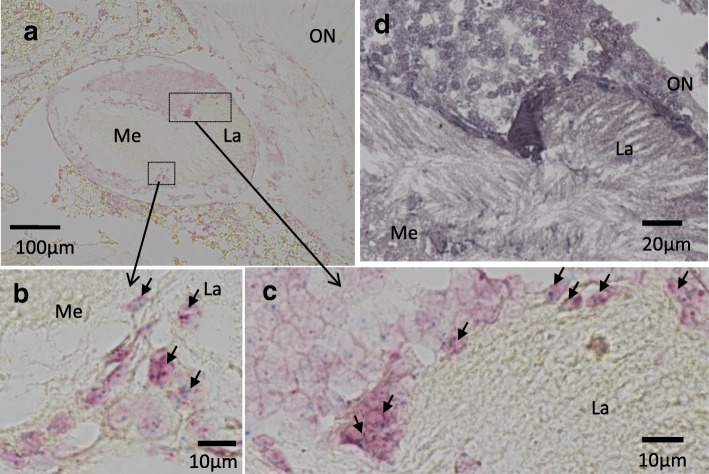
Expression of *Gb’per*, *Gb’cry2*, and *Gb’c-fosB* in the optic lobe of the cricket *Gryllus bimaculatus*. **a-c** In situ hybridization of *Gb’per* (red) and *Gb’cry2* (blue) in the optic lobe sampled at ZT18. **b** and **c** shows magnification of areas near outer chiasma indicated in **a**. Short arrows indicate the cells coexpressing *Gb’per* and *Gb’cry2*. **d** In situ PCR of *Gb’c-fosB* in the optic lobe after 1 h light exposure at ZT20. *Gb’c-fosB* was strongly expressed in the cells near outer chiasma and along the outer surface of lamina. For further explanations see text

To determine whether *Gb’cry2*, *Gb’per*, and *Gb’c-fosB* are expressed in the clock neurons, we examined their expression in the optic lobe by in situ hybridization or in situ RT-PCR. Although *Gb’per* and *Gb’cry2* were expressed in many cells in the optic lobe at ZT18, they co-localized in some neurons that are located near the outer chiasma between the lamina and medulla and the outer limb of lamina (Fig. 5). By in situ RT-PCR *Gb’c-fosB* was found to be expressed by 1 h light exposure at ZT20 in cells closely located near the area in which *Gb’per* and *Gb’cry2* are expressed. These results suggest that *Gb’per*, *Gb’cry2* and *Gb’c-fosB* are co-expressed, at least in some clock neurons.

## Discussion

### *Gb’cry1* and *Gb’cry2* play separate roles in photic entrainment

In insects, *cry1* and *cry2* are thought to encode a photoreceptor involved in photic entrainment and a transcriptional repressor in the clock oscillatory mechanism, respectively [14, 29–31]. However, we have recently shown that both *Gb’cry1* and *Gb’cry2* form an oscillatory loop that can operate independently of the *Gb’per*/*Gb’tim* loop in *G. bimaculatus* [23]. In the present study, we have clearly shown that *Gb’cry1* and *Gb’cry2* are both involved in the photic entrainment pathway of the cricket’s circadian clock in a manner different from that of *Drosophila* [11]. Double knock-down of *Gb’cry1* and *Gb’cry2* severely disrupted re-entrainment to shifted light cycles, with some crickets losing entrainability and others attaining longer transient cycles (Fig. 1, Table 2). The *Gb’cry*-dependent entrainment pathway is apparently different from the transcription-dependent entrainment pathway, or *Gb’Pdp1* pathway, which includes *Gb’Pdp1* as the first responder and works only when the light phase is extended [22]. The two *Gb’cry* genes most likely play different roles, since the effect of double RNAi was not a simple combination of the single knock-down effect for each of these genes.

The *Gb’cry2* knock-down accelerated re-synchronization of the locomotor rhythm to delayed LDs (Fig. 1c). Although we still need to examine whether this is true entrainment, this fact is reminiscent of the report that *cry* knock-out mice have significantly larger phase delays than wild-type mice [32]. Because *Gb’cry2* is an essential component of the *cry*-oscillatory loop [23], its knock-down results in a severe impairment of the loop. The acceleration of light resetting may be caused by the *Gb’Pdp1* pathway, which is still functional even in this condition. However, entrainment through the *Gb’Pdp1* pathway apparently incomplete, because double RNAi of *Gb’cry* genes prevents photic entrainment. This may also partly explain the large Ψ of the rhythm after phase shifts in *Gb’cry* double RNAi crickets (Table 1), which is similar to *cry*-deficient mice [32]. In contrast, *Gb’cry1*^RNAi^ only has a small effect on entrainment (Fig. 1b), because the *cry*-loop can be operated by *Gb’cry2* variants [23].

### The *Gb’c-fosB* pathway is involved in photic entrainment

**Fig. 6:**
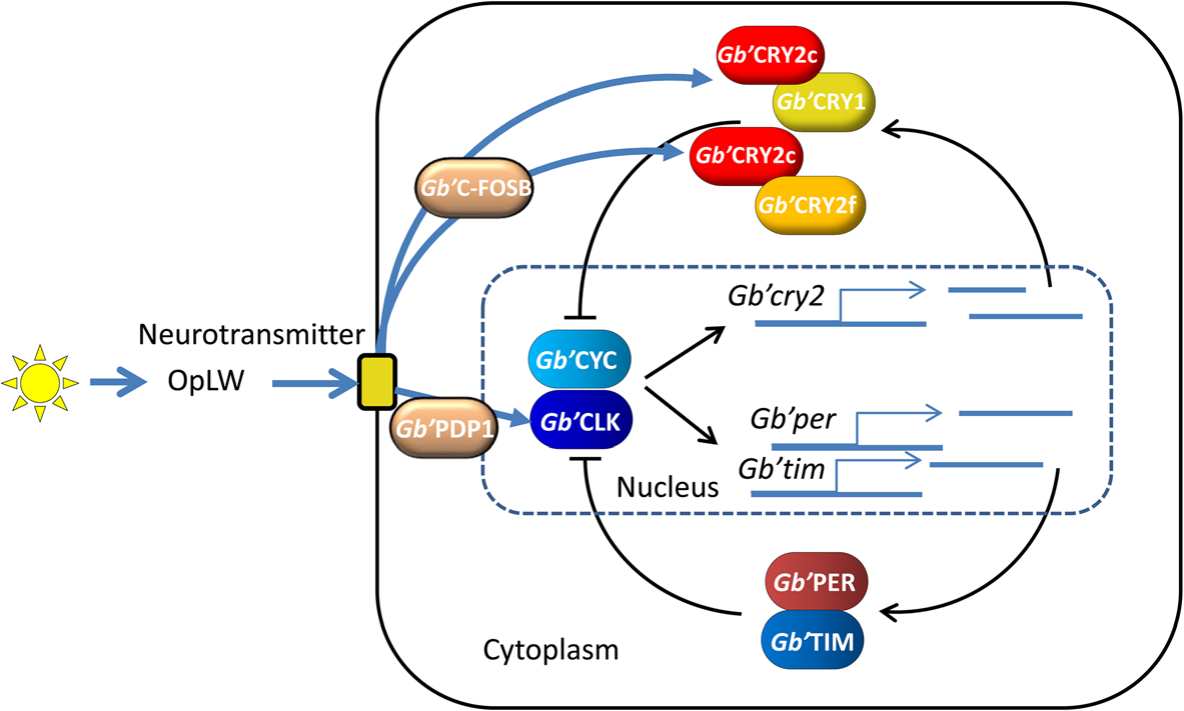
A model for the light entrainment mechanism of the cricket circadian clock. The clock includes two major oscillatory loops, one for *Gb’per* and *Gb’tim,* like in *Drosophila,* and the other for *Gb’cry2*. The latter is comprised of *Gb’cry1* and two *Gb’cry2* isoforms, *Gb’cry2c* and *Gb’cry2f*, and their product proteins form a complex that suppresses the transcription mediated by *Gb’*CLK/*Gb’*CYC complex [23]. Light is perceived by the retinular cells in the compound eye expressing *Gb’opLW*, and the information is transmitted to the clock neuron in the optic lobe through neurotransmitters. This neurotransmission causes upregulation of *Gb’c-fosB* in the clock neurons, which finally affects the *Gb’cry2* oscillatory loop. The change in the *Gb’cry2* loop may reset the whole clock system since the *Gb’cry2* loop interact with the *Gb’per/Gb’tim* loop by influencing *Gb’*CLK/*Gb’*CYC. In addition to the *Gb’cry2* pathway, the *Gb’Pdp1* pathway resets the clock by upregulating *Gb’Clk* expression when light off was delayed [22]

Although the molecular mechanism of *Gb’cry*-dependent light resetting of the cricket’s clock still remains to be explored, *Gb’c-fosB* apparently plays a regulatory role upstream of *Gb’cry*s since *Gb’c-fosB* expression was upregulated by light even after knocking-down of *Gb’cry*s (Fig. 4)*.* This hypothesis is strongly supported by the finding that *Gb’c-fosB* expression detected by in situ RT-PCR overlapped with that of cells co-expressing *Gb’per* and *Gb’cry2. c-fos* has been used as a marker for light input in vertebrate circadian clocks because of its rapid induction after light exposure [33]. Its product protein forms a complex, activating protein 1 (AP-1), with other transcription factor genes, such as *jun* gene family members, and activates transcription of target genes [34]. However, its role in resetting of the circadian clock remains mostly unclear. Our results revealed for the first time that *c-fos* plays a major role in resetting the clock in insects, since *Gb’c-fo*s^RNAi^ strongly prevents light induced phase shifts of the circadian rhythm. This also suggests that *Gb’cry*s are downstream of *Gb’c-fosB*. At present, although the connection between *Gb’c-fosB* and *Gb’cry*s is unclear, F-box and leucin rich repeat proteins (FBXL) and Bromodomain and WD repeat domain containing 3 (BRWD3) may be mediating this connection, as they are known to regulate CRY degradation by ubiquitination of CRY [35–37]. Light input to the *Gb’c-fosB* pathway is through *Gb’*OpLW, because the light-induced upregulation of *Gb’c-fosB* is eliminated by *Gb’opLW*^RNAi^. Based on the present findings and those of previous studies, the most likely hypothesis appears to be that light information is supplied to the clock neurons by neurotransmission through the OpLW pathway, causing induction of *Gb’c-fosB*, followed by a functional modulation of FBXL or BRWD3, which may ubiquitinate CRYs and reset the oscillatory mechanism including CRYs (Fig. 6). This hypothesis should be tested in future studies.

Besides the *Gb’c-fosB* mediated pathway, *Gb’cry*s might also be regulated by light through other mechanisms. Our previous study showed that light phase extension at early night induced *Gb’cry2* upregulation with an increase of *Gb’Pdp1* and *Gb’Clk* in nymphal crickets [22], suggesting the E-box mediated transcription of *Gb’cry2* by *Gb’Clk*. Another possibility is through a D-box mediated regulation known for zebrafish clocks, in which light induces *cry1a* expression through a D-box mediated mechanism including PAR bZip factors, PAR and TEF-1 [38]. Because D-boxes are found in the cis-regulatory region of *Gb’cry1* and *Gb’cry2*, a similar mechanism might be involved in the cricket clock. These issues should be addressed in future studies.

### Heterogeneous nature of clock cells

In the cricket’s clock, there must be at least two sets of clock neurons since some fraction of the *Gb’cry1/Gb’cry2* double RNAi crickets showed a rhythm dissociation into two components when the light cycle was shifted by 6 h: one component still retained photic entrainability, while it was lost in the other (Fig. 1e). The heterogenous cellular organization of the clock is quite similar to that observed in *Drosophila* and mammals [39, 40], where entrainability to light is different between the cerebral or SCN clock neurons [41–43].

The heterogeneous cellular nature of the clock may explain the effect of *Gb’cry2*^RNAi^ on the free-running locomotor rhythm. We have previously shown that *Gb’cry2*^RNAi^ crickets have a wide variety of free-running periods in DD [23], which may reflect the underlying oscillatory neurons. The lack of *Gb’*CRY2 following RNAi may destroy the stable free-running of the clock neuron, hence causing dissociation among the cells. This is reminiscent of the mammalian SCN clock, where CRY plays a role as a coupling factor of clock cells that have variable free-running periods [39, 44–46].

### Comparison with other insect clocks

In the present study, we found for the first time that both *Gb’cry1* and *Gb’cry2* play important roles in photic entrainment of the circadian clock in crickets. In hemimetabolous insects, the photoreceptor for entrainment is believed to reside in the compound eye [19, 47, 48], and this assumption has been confirmed at the molecular level in the cricket *G. bimaculatus* [21]. Therefore, *Gb’cry1* and *Gb’cry2* are both active in the clock resetting mechanism downstream of the neurotransmission from the retinal photoreceptor. Although the exact role of *Gb’cry* genes should be examined in future studies, the cricket’s *Gb’*CRY1 likely works together with *Gb’*CRY2 to form an oscillatory feedback loop, which can operate independently of the *Gb’per*/*Gb’tim* loop [23]. As for insect *cry2*, it is believed to be involved in the clock machinery as a clock component [14]. However, we have shown for the first time that *Gb’cry2* also plays an important role in the photic entrainment of the clock. Our results shed light on the insect clock mechanism and provide deeper understanding of its photic entrainment and diversification.

## Conclusions

Photic entrainment is an essential property of the animal circadian clock that sets the appropriate timing of behavioral and physiological events in a 24 h cycle. Although most insects use compound eyes as photoreceptors for entrainment, the molecular mechanisms underlying entrainment remain largely unknown. Here we elucidated for the first time the molecular entrainment pathway using a hemimetabolous insect, the cricket *Gryllus bimaculatus*. Our results suggest that neural signals mediated by green-sensitive opsins in the compound eye first up-regulate an isoform of the bZip transcription factor gene, *Gb’c-fos*, which subsequently resets the clock through *Gb’cry1* and *Gb’cry2*. These findings contribute to understanding of the photic entrainment mechanism of insect clocks.

## Additional files

Additional file 1: **Table S1.** PCR primers used for quantitative RT-PCR and dsRNA synthesis. The primers tagged with T7 or T3 promoter sequences were used for PCR amplification for dsRNA synthesis. T7 and T3 sequences are underlined. (DOCX 18 kb)

Additional file 2: **Figure S1.** Effect of RNAi of *cry* genes on locomotor activity during the first 3 h after light-on in the cricket *Gryllus bimaculatus*. The activity was measured on the first day after 6 h phase advance of light-on. Error bars indicate SEM. Numbers in parenthesis indicate the number of animals used. *Gb’cry2*^RNAi^ and *Gb’cry1*^RNAi^/*Gb’cry2*^RNAi^ significantly reduced the light-induced locomotor activity compared to *DsRed2*^RNAi^ treatment (*P < 0.05, **P < 0.01, Dunnett’s test). (PDF 58 kb)

Additional file 3: **Figure S2.** *Gb’c-fos*^RNAi^ significantly down-regulated both *Gb’c-fosA* and *Gb’c-fosB* mRNA levels in the optic lobe of the cricket *Gryllus bimaculatus* (**P < 0.01, t-test). The optic lobes were collected at ZT20 seven days after dsRNA injection. mRNA levels were measured by qPCR and are shown relative to those of *Gb’rpl18a*. The values shown are mean ± SEM of six samples. (PDF 49 kb)

Additional file 4: **Figure S3.** A: *Gb’c-fosA*^RNAi^ had no significant effects on the light induced phase advance in the cricket *Gryllus bimaculatus*. A 3 h light pulse was given at ZT20 on the day of transfer to DD, which was seven days after dsRNA injection. Numbers in the parenthesis indicate the number of animals used. B and C: *Gb’c-fosA*^RNAi^ significantly knocked down *Gb’c-fosA* mRNA levels (*P < 0.05, t-test), but had no significant effect on *Gb’c-fosB* mRNA levels. mRNA levels were measured by qPCR and are shown relative to those of *Gb’rpl18a*. The values shown are mean ± SEM of four samples. (PDF 69 kb)
