# Supplementary material for: A novel photic entrainment mechanism for the circadian clock in an insect: involvement of c-fos and cryptochromes
Source: Zoological Lett. 2018 Sep 18;4:26. doi: 10.1186/s40851-018-0109-8 (PMC6145112; doi:10.1186/s40851-018-0109-8)
Supplement: Supplementary file 1 — Table S1. PCR primers used for quantitative RT-PCR and dsRNA synthesis. The primers tagged with T7 or T3 promoter sequences were used for PCR amplification for dsRNA synthesis. T7 and T3 sequences are underlined. (DOCX 18 kb) [file 40851_2018_109_MOESM1_ESM.docx]

Table S1. PCR primers used for quantitative RT-PCR and dsRNA synthesis. The primers tagged with T7 or T3 promoter sequences were used for PCR amplification for dsRNA synthesis. T7 and T3 sequences are underlined.

| Genes | Forward | Reverse |
| --- | --- | --- |
| Quantitative RT-PCR | |  |
| *Gb’cry1* | 5’-TTGAGCAGGATTGTGAACCA-3’ | 5’-GTGGTGGTACACCTCCATTTG-3’ |
| *Gb’cry2* | 5’-TTCGACACGGAAGGTCTGCT-3’ | 5’-CGGCCAAATGATGCTACCC-3’ |
| *Gb’c-fosA* | 5’-CCTGCCTTCATCTGCGTACG-3’ | 5’-GTCTCACTGGGCGAAACGTG-3’ |
| *Gb’c-fosB* | 5’-GGCGGCTTGTGTGTTTGTG-3’ | 5’-CCCGAATTGAGGCCGTCC-3’ |
| *Gb’Pdp1* | 5’-TCCCGACGACAAGAAGGAG-3’ | 5’-AGCGTCTTGTCCCAGAGGTTG-3’ |
| *Gb’rpl18a* | 5’-GCTCCGGATTACATCGTTGC-3’ | 5’-GCCAAATGCCGAAGTTCTTG-3’ |
| dsRNA synthesis | |  |
| *Gb’cry1* | 5’-TAATACGACTCACTATAGGGTGGGAACAAGGAAAGACTGG-3’ | 5’-AATTAACCCTCACTAAAGGGCGACAATGAGGTGGAGGATT-3’ |
| *Gb’cry2* | 5’-TAATACGACTCACTATAGGGCTGCGACAAATAACCCCAAC-3’ | 5’-AATTAACCCTCACTAAAGGGCTCTCAGGAGCATTCCAAGG-3’ |
| *Gb’opLW* | 5’-AATTAACCCTCACTAAAGGGCGTGCTGGGAGTGATCT-3’ | 5’-TAATACGACTCACTATAGG GCCACGTCTTGGTCAGGTAG-3’ |
| *Gb’opBlue* | 5’-TAATACGACTCACTATAGG TGGTATTGGTTCTGCCATCA-3’ | 5’-TAATACGACTCACTATAGG ATTGCCACAGTAGCATAAGG-3’ |
| *Gb’c-fosA* | 5’-TAATACGACTCACTATAGGGCCTGCCTTCATCTGCGTACG-3’ | 5’-TAATACGACTCACTATAGGGGTCTCACTGGGCGAAACGTG-3’ |
| *Gb’c-fos* | 5’-TAATACGACTCACTATAGGGGGGATGTCACCAGAGGAAGA-3’ | 5’-TAATACGACTCACTATAGGGGGAGGTTTTGCACCACTTGT-3’ |
| *DsRed2* | 5’-TAATACGACTCACTATAGGGTCATCACCGAGTTCATGCG-3’ | 5’-TAATACGACTCACTATAGGGCTACAGGAACAGGTGGTGGC-3’ |
