# Supplementary material for: A novel photic entrainment mechanism for the circadian clock in an insect: involvement of c-fos and cryptochromes
Source: Zoological Lett. 2018 Sep 18;4:26. doi: 10.1186/s40851-018-0109-8 (PMC6145112; doi:10.1186/s40851-018-0109-8)
Supplement: Supplementary file 2 — Figure S1. Effect of RNAi of cry genes on locomotor activity during the first 3 h after light-on in the cricket Gryllus bimaculatus. The activity was measured on the first day after 6 h phase advance of light-on. Error bars indicate SEM. Numbers in parenthesis indicate the number of animals used. Gb’cry2RNAi and Gb’cry1RNAi/Gb’cry2RNAi significantly reduced the light-induced locomotor activity compared to DsRed2RNAi treatment (*P < 0.05, **P < 0.01, Dunnett’s test). (PDF 58 kb) [file 40851_2018_109_MOESM2_ESM.pdf]

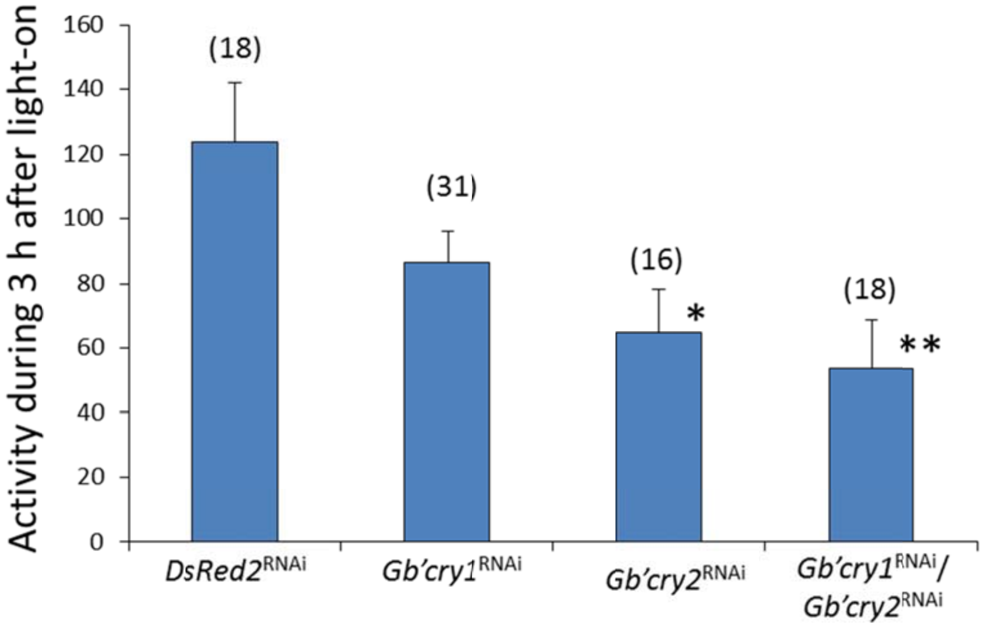

Figure S1. Effect of RNAi of *cry* genes on locomotor activity during the first 3 h after light-on in the cricket *Gryllus bimaculatus*. The activity was measured on the first day after 6 h phase advance of light-on. Error bars indicate SEM. Numbers in parenthesis indicate the number of animals used. *Gb'cry2*^{RNAi} and *Gb'cry1*^{RNAi}/*Gb'cry2*^{RNAi} significantly reduced the light-induced locomotor activity compared to *DsRed2*^{RNAi} treatment (*P<0.05, **P<0.01, Dunnett's test).
