# Supplementary material for: A novel photic entrainment mechanism for the circadian clock in an insect: involvement of c-fos and cryptochromes
Source: Zoological Lett. 2018 Sep 18;4:26. doi: 10.1186/s40851-018-0109-8 (PMC6145112; doi:10.1186/s40851-018-0109-8)
Supplement: Supplementary file 3 — Figure S2. Gb’c-fosRNAi significantly down-regulated both Gb’c-fosA and Gb’c-fosB mRNA levels in the optic lobe of the cricket Gryllus bimaculatus (**P < 0.01, t-test). The optic lobes were collected at ZT20 seven days after dsRNA injection. mRNA levels were measured by qPCR and are shown relative to those of Gb’rpl18a. The values shown are mean ± SEM of six samples. (PDF 49 kb) [file 40851_2018_109_MOESM3_ESM.pdf]

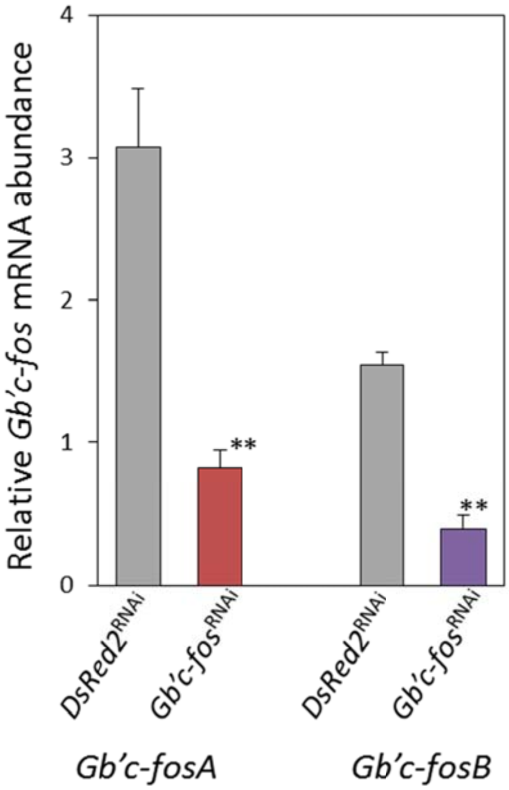


**Figure S2.** *Gb'c-fos*$^{RNAi}$ significantly down-regulated both *Gb'c-fosA* and *Gb'c-fosB* mRNA levels in the optic lobe of the cricket *Gryllus bimaculatus* (**P<0.01, t-test). The optic lobes were collected at ZT20 7 days after the dsRNA injection. mRNA levels were measured by qPCR and are shown relative to those of *Gb'rpl18a*. The values shown are mean±SEM of 6 samples.
