# Supplementary material for: A novel photic entrainment mechanism for the circadian clock in an insect: involvement of c-fos and cryptochromes
Source: Zoological Lett. 2018 Sep 18;4:26. doi: 10.1186/s40851-018-0109-8 (PMC6145112; doi:10.1186/s40851-018-0109-8)
Supplement: Supplementary file 4 — Figure S3. A: Gb’c-fosARNAi had no significant effects on the light induced phase advance in the cricket Gryllus bimaculatus. A 3 h light pulse was given at ZT20 on the day of transfer to DD, which was seven days after dsRNA injection. Numbers in the parenthesis indicate the number of animals used. B and C: Gb’c-fosARNAi significantly knocked down Gb’c-fosA mRNA levels (*P < 0.05, t-test), but had no significant effect on Gb’c-fosB mRNA levels. mRNA levels were measured by qPCR and are shown relative to those of Gb’rpl18a. The values shown are mean ± SEM of four samples. (PDF 69 kb) [file 40851_2018_109_MOESM4_ESM.pdf]

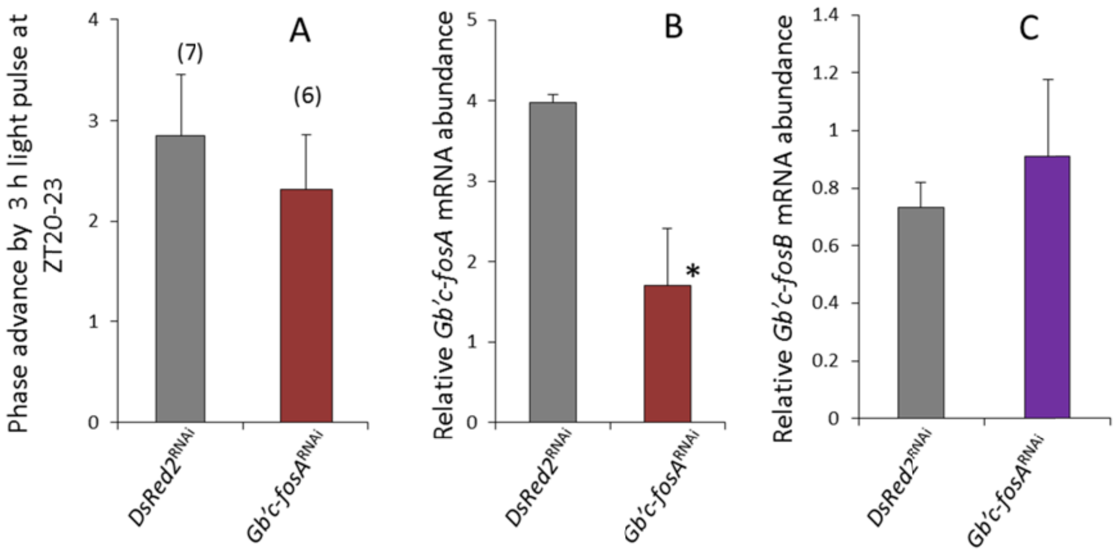

Figure S3. A: *Gb'c-fosA*^{RNAi} had no significant effects on the light induced phase advance in the cricket *Gryllus bimaculatus*. A 3 h light pulse was given at ZT20 on the day of transfer to DD, which was 7 days after dsRNA injection. Numbers in the parenthesis indicate the number of animals used. B and C: *Gb'c-fosA*^{RNAi} significantly knocked down *Gb'c-fosA* mRNA levels (*P<0.05, t-test), but had no significant effect on *Gb'c-fosB* mRNA levels. mRNA levels were measured by qPCR and are shown relative to those of *Gb'rpl18a*. The values shown are mean±SEM of 4 samples.
